# Can the cGAS-STING Pathway Play a Role in the Dry Eye?

**DOI:** 10.3389/fimmu.2022.929230

**Published:** 2022-06-24

**Authors:** Weijie Ouyang, Shoubi Wang, Jiaoyue Hu, Zuguo Liu

**Affiliations:** ^1^ Eye Institute of Xiamen University, Xiamen University, Xiamen, China; ^2^ Fujian Provincial Key Laboratory of Ophthalmology and Visual Science, Xiamen University, Xiamen, China; ^3^ Fujian Engineering and Research Center of Eye Regenerative Medicine, Xiamen University, Xiamen, China; ^4^ School of Medicine, Xiamen University, Xiamen, China; ^5^ Department of Ophthalmology, Xiang’an Hospital of Xiamen University, Xiamen University, Xiamen, China; ^6^ Department of Endocrinology and Diabetes, Xiamen Diabetes Institute, Xiamen University, Xiamen, China; ^7^ Xiamen Clinical Medical Center for Endocrine and Metabolic Diseases, Xiamen University, Xiamen, China; ^8^ Xiamen Diabetes Prevention and Treatment Center, Xiamen University, Xiamen, China; ^9^ Fujian Key Laboratory of Diabetes Translational Medicine, The First Affiliated Hospital of Xiamen University, School of Medicine, Xiamen University, Xiamen, China; ^10^ Xiamen University Affiliated Xiamen Eye Center, Xiamen, China; ^11^ Department of Ophthalmology, The First Affiliated Hospital of University of South China, Hengyang, China

**Keywords:** cGAS-STING pathway, dry eye, dsDNA, inflammation, innate immunity

## Abstract

Dry eye is one of the most common ocular surface diseases in the world and seriously affects the quality of life of patients. As an immune-related disease, the mechanism of dry eye has still not been fully elucidated. The cGAS-STING pathway is a recently discovered pathway that plays an important role in autoimmune and inflammatory diseases by recognizing dsDNA. As an important signal to initiate inflammation, the release of dsDNA is associated with dry eye. Herein, we focused on the pathophysiology of the immune-inflammatory response in the pathogenesis of dry eye, attempted to gain insight into the involvement of dsDNA in the dry eye immune response, and investigated the mechanism of the cGAS-STING pathway involved in the immune-inflammatory response. We further proposed that the cGAS-STING pathway may participate in dry eye as a new mechanism linking dry eye and the immune-inflammatory response, thus providing a new direction for the mechanistic exploration of dry eye.

## Introduction

Dry eye is a multifactorial ocular surface disease that is characterized by the loss of tear film homeostasis and accompanied by ocular discomfort (1). Tear film instability, hyperosmotic stress, ocular surface inflammation, and neurosensory abnormalities are the important causes of dry eye (1). According to the Dry Eye Work Shop II (DEWS II), the global prevalence of dry eye ranges from 5-50% (2), and the discomfort caused by dry eye usually leads to a decrease in quality of life companied by high incidence of depression and anxiety (3), suggesting that dry eye is becoming a global public health issue. Inflammation plays a vital role in the pathophysiology of dry eye, but the mechanisms remain unclear.

The inflammatory response is an important pathological feature of dry eye. The expression of inflammatory factors (4) such as IL-1β, IL-6, IL-8, TNF-a and IFN-γ and infiltrated inflammatory cells (5, 6), has been shown to be increased on the ocular surface of dry eye patients. Some inflammatory signaling pathways have been reported to be involved in the pathogenesis of dry eye (7). For example, hyperosmotic stress activates the NF-κB signaling pathway, which mediates inflammatory cytokine release and adaptive immune activation (8). Therefore, anti-inflammatory therapy [e.g., treatment with cyclosporine A (9, 10)] is an important treatment for dry eye. However, there are still some dry eye patients who have no significant effect on existing treatments (9). Therefore, new possible mechanisms need to be considered to provide new possibilities for dry eye treatment.

The cGAS-STING signaling pathway is a recently discovered inflammatory signaling pathway (11), which has become a hot topic in various immune and inflammatory diseases due to its ability to recognize dsDNA and to mediate the inflammatory response (12). In addition, dsDNA recognition and activation of inflammatory signaling pathways have also been reported to be associated with dry eye (13). Reduced DNA enzyme content and dsDNA accumulation have been detected in the tears of dry eye patients (14). Furthermore, hyperosmotic stress activates the NLRP3-IL-1β signaling pathway through dsDNA oxidative damage (13, 15). This suggests that dsDNA could play a role in the pathogenesis of dry eye. Therefore, in this review, we mainly focused on the mechanism of dry eye inflammation and the mechanism of the cGAS-STING signaling pathway in mediating the immune inflammatory response; additionally, we summarized the mechanism of dsDNA damage and release, as well as the mediation of the inflammatory response in a hypertonic environment. Finally, we discussed the possibility of the cGAS-STING pathway as a new mechanism in dry eye.

## The Mechanisms of Immunoinflammatory Responses in Dry Eye

### The Vicious Cycle of Inflammation in Dry Eye

The immune-inflammatory response is the core pathophysiology of dry eye. The epithelial cells of the ocular surface can be damaged by tear hypertonicity, thus leading to the release of a large number of proinflammatory factors, such as IL-1β, IL-6, and TNF-α. And then, dendritic cells recognize the inflammatory cytokines and migrate to drainage lymph nodes of the neck through lymphatic vessels of the ocular surface. Dendritic cells further bind with T cells to promote the differentiation of T cells into Th1 and Th17 cells. The differentiated T cells enter the blood and reflux to the ocular surface, after which they secrete inflammatory factors such as INF-γ and IL-17, further leading to epithelial cell damage. Moreover, the damage to epithelial cells further promotes the release of inflammatory cytokines, thus forming a vicious cycle of “injury-inflammation”, which ultimately results in chronic and persistent inflammation. In addition, this vicious cycle provides an entry point for any of the factors that can cause dry eye, thus explaining the diversity of factors in the pathogenesis of dry eye (7). Regardless of the cause, the core of the pathogenesis of dry eye is attributed to immune inflammatory responses.

### The NF-κB Signaling Pathway Is the Key Target Mediating Innate and Adaptive Immunity in Dry Eye

**Figure 1:**
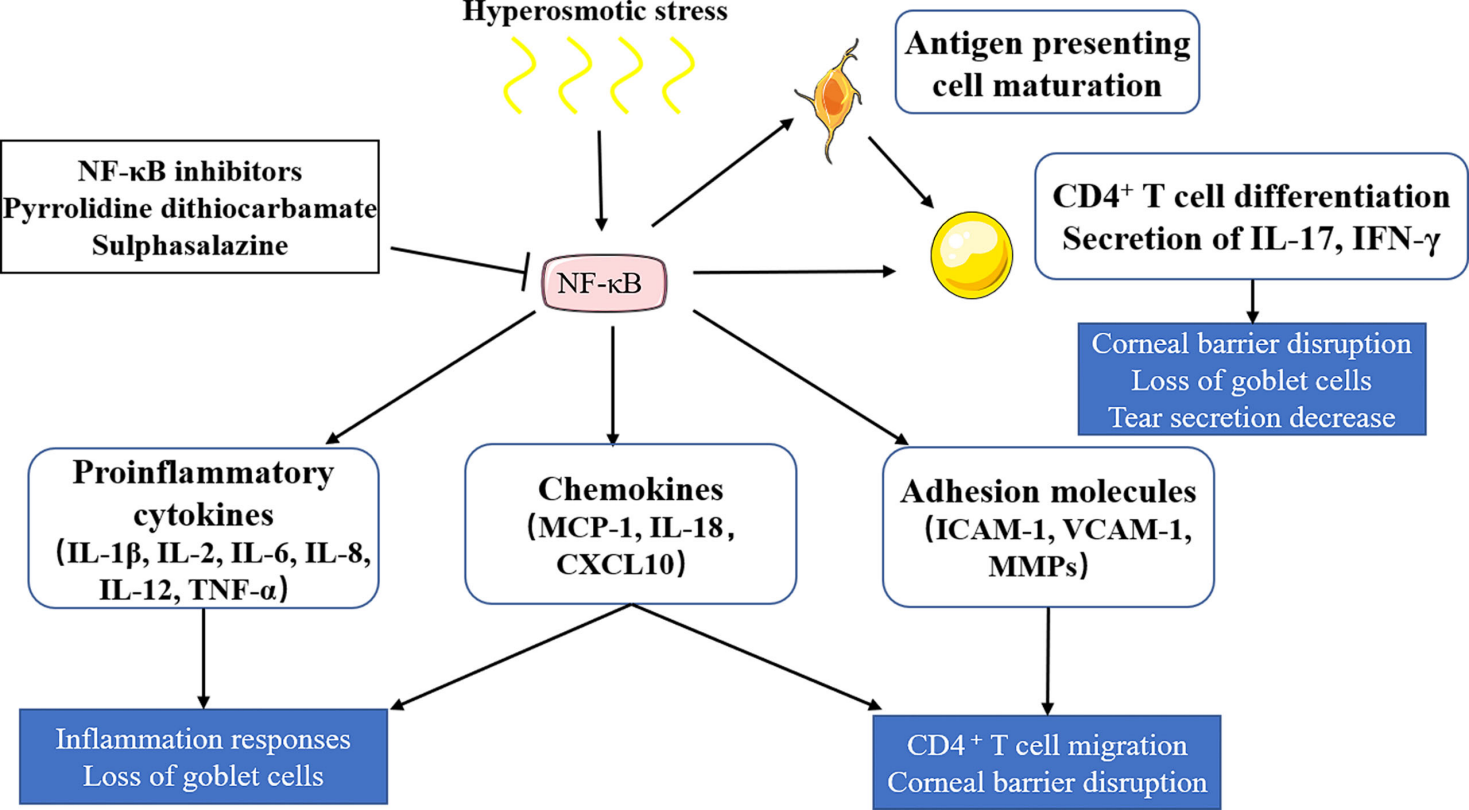
The role of the NF-κB signaling pathway in dry eye. Hyperosmotic stress induces the activation of the NF-κB signaling pathway, which induces the releases of proinflammatory cytokines, chemokines and adhesion molecules, and activates antigen-presenting cells and CD4+ T cells. Proinflammatory cytokines (IL-1β, IL-2, IL-6, IL-8, IL-12, TNF-α) and chemokines (MCP-1, IL-18, CXCL10) can lead to inflammatory responses and goblet cell loss. Chemokines and adhesion molecules (ICAM-1, VCAM-1, MMPs) can induce T cell migration and corneal barrier disruption. CD4+ T cells could be differentiated into T help cells (T_H_1 and T_H_17), which then release IL-17 and IFN-γ, leading to tear secretion reduction, corneal epithelial damage, and goblet cell decrease. NF‐κB inhibitors, including pyrrolidine dithiocarbamate and sulphasalazine, show therapeutic potential in dry eye by inhibiting NF‐κB activation.

Many signaling pathways have been reported to be involved in immune inflammatory responses of dry eye, among which the NF-κB signaling pathway is the most studied, which has been confirmed in various dry eye models (16–19). The recognized role of the NF-κB signaling pathway is to regulate innate and adaptive immunity. The NF-κB signaling pathway induces the transcription of proinflammatory cytokines (IL-1β, IL-2, IL-6, IL-8, IL-12, and TNF-α, among other factors.), chemokines (MCP-1, IL-18, and CXCL10, among other factors), and adhesion molecules (ICAM-1, VCAM-1, and MMPs, among other molecules) in different types of innate immune cells. These inflammatory mediators can not only directly participate in the induction of inflammation but also play an indirect role by promoting the differentiation of inflammatory T cells (20). The NF-κB signaling pathway can be activated by tear film instability and increased tear osmotic pressure (2), which induces downstream inflammatory factors (IL-1β, TNF-α, and IL-6) and activates antigen-presenting cells and CD4+ T cells to release IL-17 and IFN-γ, thus leading to reduced tear secretion, corneal epithelial damage, and decreased goblet cells (21). Once the NF-κB signaling pathway is inhibited, the expression of IL-1β, TNF-α, and IL-6 on the ocular surface of dry eye, as well as the activation of immune cells, can be down-regulated (22). In conclusion, the NF-κB signaling pathway plays an important role in dry eye. (**Figure 1**)

The activated NF-κB signaling pathway and downstream inflammatory response in dry eye are not induced by infectious pathogens (7), but may be by the endogenous substance (23). One way to activate inflammatory response is the cGAS-STING pathway, which recognizes the endogenous substance to induce NF-κB signaling pathway.

## The cGAS-STING Pathway Contributes to Autoimmune and Inflammatory Diseases

### Induction of the cGAS-STING Pathway

cGAS (24) and STING (25) were first discovered in 2008 and 2013, respectively. The cGAS-STING pathway can recognize cytoplasmic dsDNA to activate the innate immune response (12). The C-terminus of cGAS contains a nucleotide transferase domain with surface grooves on the back (26). The main chain of sugar-phosphate on dsDNA interacts with the positively charged residues in the grooves on the surface of cGAS, which changes the conformation of cGAS and activates it (27, 28). This binding mode explains why cGAS does not require sequence specificity to recognize dsDNA; thus, cGAS can sense dsDNA from cytoplasmic viruses and bacteria, as well as autologous dsDNA. Activated cGAS converts adenosine 5-triphosphate (ATP) and guanosine 5-triphosphate (GTP) into cyclic GMP-AMP (cGAMP), after which cGAMP acts as a secondary messenger to bind and activate STING (29, 30). STING is located in the endoplasmic reticulum in a dimeric form, the two C-termini of which form a V-shape and contain the binding site of cGAMP (31). The conformation of STING changes after binding with cGAMP, and STING then migrates from the endoplasmic reticulum to the Golgi apparatus (32), which is necessary to induce the transcription of type I interferons (33). Furthermore, STING recruits TBK1 and IRF3 after activation, and IRF3 is phosphorylated to form a dimer and is ectopic to the nucleus, thus driving the expression of type I interferons (33, 34). Additionally, STING combines with TBK1 and IKK to phosphorylate IκB and to activate NF-κB to become ectopic to the nucleus (35, 36). Finally, the cGAS-STING pathway mediates immune-inflammatory responses through IFN and NF-κB.

### The cGAS-STING Pathway Is Involved in Autoimmune Disease

By sensing dsDNA, the cGAS-STING pathway has become a key pathway in autoimmune and inflammatory diseases (**Table 1**), such as Sjogren’s syndrome (52), systemic lupus erythematosus (SLE) (45), and multiple sclerosis (53). As a downstream target of the cGAS-STING pathway, type I interferon serves as a marker and potential therapeutic target of systemic autoimmune diseases (54, 55). TREX1 is a cytoplasmic DNA exonuclease that degrades accumulated DNA in cells (56). TREX1 mutations can mediate SLE-like pathological changes (57) with increased type I interferons, whereas the phenotypes of SLE in Trex1-/- mice depends on the activation of the cGAS-STING pathway (58). A large cohort study found that the TREX1 polymorphism is a sign of SLE susceptibility, which further strengthens the association between cGAS and SLE (59). In addition, the serum autologous DNA level in SLE patients was increased (60), the peripheral blood cGAMP concentration of approximately 15% of SLE patients was increased, and the cGAS level was also higher than that of the control group (61). Similarly, Sjogren’s syndrome is a common autoimmune disease characterized by lymphocytic infiltration and inflammation of the exocrine glands, resulting in decreased secretion of involved glands that manifests mostly as dry eye and dry mouth (62). The innate immune response mediated by type I interferons plays an important role in Sjogren’s syndrome (63, 64). The loss of the type I interferon receptor can prevent the pathological changes of Sjogren’s syndrome (65). DNA recognition by DNA receptors is the main triggering factor for the activation of type I interferons, and the activation of the cGAS-STING pathway can promote type I interferons expression by recognizing cytoplasmic DNA (12). *In vivo*, activation of the cGAS-STING pathway can mediate Sjogren’s syndrome-like pathological changes in salivary glands and lungs (66, 67). Research on the cGAS-STING pathway in Sjogren’s syndrome is still in the preliminary stage, and more evidence is needed to prove its role in Sjogren’s syndrome, thus suggesting that the cGAS-STING pathway may become a hotspot for future studies on the innate immune response.

**Table 1:** Inflammation disease links to cGAS-STING pathway.

Disease	Gene	Inflammation	Improved by cGAS inhibited or knock out	Improved by STING inhibited or knock out	Ref.
Age-related macular degeneration	Alu RNA	IFN-β, IL-18, caspase-4, caspase-11		Yes	(37)
*Aspergillus fumigatus* keratitis		TBK1, IFN-β, IL-1β, IL-6	Yes		(38)
Acute kidney injury	BAX	IL-6, ICAM1, CXCL10, GM-CSF, NF-κB	Yes	Yes	(39)
Kidney fibrosis	TFAM	IL-1β, CCL2, IL-6, TNF-α, NF-κB	Yes	Yes	(40)
Parkinson’s disease	Parkin/PINK1	IL-6, IFNβ1, TNFα, IL-1β, CCL2, IL-12(p70), IL-13, IL-17, CXCL1, CCL4		Yes	(41)
Myocardial infarction		IRF3, IFNβ1, CXCL10, IL-6, TNFα, IL-1β,	Yes	Yes	(42)
Aicardi–Goutieres syndrome	TREX1, RNase H2	CXCL10, ISGs, MX1, IFN-β1	Yes	Yes	(43, 44)
systemic lupus erythematosus	TREX1, DNase I, DNase IL3, Fcgr2b	IFN-γ, IFN-β, CXCL10, IL-1α, IL-6, TNF-α		Yes	(45–47)
Alcoholic liver disease		IFNβ1, IRF3, TBK1		Yes	(48)
Nonalcoholic steatohepatitis		TNF-α, IL-6, IL-1β, NF-κB		Yes	(49)
STING-associated vasculopathy with onset in infancy (SAVI)		IFN-α, IFN-β, ISGs, STAT1			(50, 51)

### The cGAS-STING Pathway Is Involved in Inflammation Disease

The cGAS-STING pathway has been confirmed not only to be involved in autoimmune diseases but also to mediate inflammatory responses (33). STING-associated vasculopathy with onset in infancy (SAVI) is an autoinflammatory disease caused by gain-of-function mutations in STING (50). Gain-of-function STING mutations induces overproduction of type I interferons (IFN-α and IFN-β), leading to skin disorder, inflammatory pulmonary and liver manifestations in SAVI patients (68, 69). Many proinflammatory factors, including IL-6, TNF, IL-1β, and IFN-γ, were found to be elevated in the serum of patients with Parkinson’s disease, which is an inflammatory disease. It has been reported that damaged mitochondria can be cleared by PINK1 and Parkin *via* autophagy (70). Prkn^-/-^ or Pink1^-/-^ mice could mimic the phenotypes of Parkinson’s disease, which is accompanied by mitochondrial stress responses and the release of DAMPs, thus leading to the release of a large number of inflammatory factors through the STING-mediated IFN-I response, whereas knockout of STING reduced inflammation and improved the disease phenotype of Prkn^-/-^ or Pink1^-/-^ mice (41). Furthermore, geographical atrophy is a manifestation of age-related macular degeneration (AMD) that is characterized by DICER1 deficiency and the accumulation of endogenous Alu RNA, which could trigger the release of mitochondrial DNA to activate cGAS and further induce IFN-β and IL18, as well as drive caspase 1-, 4-, and 11-related inflammation (37). These responses ultimately lead to RPE degeneration, thus suggesting that cGAS-driven inflammasome activation is involved in AMD (37). In mouse models of ischemic myocardial infarction and drug-induced acute kidney injury, the cGAS-STING pathway was shown to be activated, thus leading to the release of a large number of inflammatory factors (71, 72). Downregulation of the cGAS-STING pathway was found to alleviate inflammation and relieve ventricular dysfunction and kidney damage (39, 42). Similarly, in chronic renal fibrosis, the cGAS-STING pathway was activated due to mitochondrial dysfunction, thus resulting in the release of inflammatory cytokines and the recruitment of immune cells, which aggravated renal fibrosis that could be improved by STING knockout (40). The abovementioned studies have indicated the role of the cGAS-STING pathway in inflammation, thus suggesting that it may become a potential target for the treatment of inflammatory diseases.

## The Role of DsDNA in Dry Eye

DNA is not only the genetic material of organisms but has also been found to be a pattern component in innate immune responses (73). When bacterial infection occurs, bacterial DNA acts as a foreign antigen to activate the host’s strong innate immune system and induce the expression of type I interferons, including IFN-α and IFN-β (74). Furthermore, autologous DNA can act as an endogenous ligand of DNA receptors, and overreleased DNA induces innate immune responses and increases serum type I interferon levels, which are involved in SLE (75, 76). Anti-ds DNA antibodies have also been detected in patients with Sjogren’s syndrome (77). These studies have dramatically changed the traditional view that DNA only acts as the genetic material of organisms (73). Therefore, we wondered whether dsDNA could participate in dry eye.

### Hyperosmotic Stress Is an Important Cause of Dry Eye and Mediate the Release of Nuclear/Mitochondrial DNA Into the Cytoplasm

In normal cells, DNA is located in the nucleus or mitochondria. Once damaged, nuclear or mitochondrial DNA is released into the cytoplasm. Increased extracellular osmotic pressure can cause damage to corneal epithelial cells (13, 78, 79), the kidney (80), and cardiomyocyte (81), among other tissues. As the core mechanism of dry eye, the increased tear osmotic pressure caused by tear film instability can injure corneal epithelial cells. The mechanisms are summarized in the following sections.

1. Hyperosmotic stress can mediate increased cellular oxidative stress. Accumulating evidence has demonstrated that increased osmotic pressure mediates the accumulation of intracellular reactive oxygen species (ROS) in human corneal epithelial cells (HCE), which further induces lipid peroxidation and increases 4-hydroxynonenal (4-HNE) and malondialdehyde (MDA) toxic products (79), thus leading to cytotoxic injury. In addition, aconitase-2 and 8-hydroxy-2 deoxyguanosine (8-OHdG) in ribosomal and mitochondrial DNA were found to be increased, whereas antioxidant enzymes, including superoxide dismutase 1 (SOD1) and glutathione peroxidase 1 (GPX1), were reduced (13, 79), thus causing oxidative damage to cellular DNA.

2. Hyperosmotic stress can cause mitochondrial dysfunction. Compared with nuclear DNA, mitochondrial DNA is susceptible to various damage factors without the protection of histones and an efficient damage repair system (82). Under hyperosmotic stress, mitochondrial nicotinamide adenine dinucleotide (NADH) diffuses into the cytoplasm, resulting in mitochondrial depolarization, an increase in the adenosine diphosphate/adenosine triphosphate (ADP/ATP) ratio, and finally mitochondrial energy metabolism disorders (83). In addition, hypertonicity has been shown to induce the high expression of mitochondrial BCL-2-like protein 4 (Bax) (83), thus leading to an increase in mitochondrial membrane permeability and the release of mitochondrial DNA into the cytoplasm (39).

3. Hyperosmotic stress can cause DNA breaks increase. In hyperosmotic stress, the chromatin nucleus shrinks, followed by cell apoptosis, which is characterized by DNA fragmentation (81).

In summary, as common causes of dry eye, hyperosmotic stress can mediate cell damage through cellular oxidative stress, mitochondrial dysfunction, and DNA scission. Damaged cytoplasmic DNA may participate in the immune response mechanism of dry eye, but more research is needed to prove this supposition.

### Oxidative MtDNA Initiates Inflammation in Dry Eye

Hyperosmotic stress causes DNA oxidative damage and initiates an inflammatory response. The most direct evidence suggests that hyperosmotic stress can cause overproduction of intracellular ROS and mtDNA damage. Oxidative mtDNA increases 8-OHdG expression, which can cause an imbalance in NLRP3/NLRP6, caspase-1 activation, and the release of IL-1β and IL-18. By using the antioxidant L-carnitine, the reduction in mtDNA damage can inhibit the release of IL-1β and IL-18 (84, 85). In addition, 8-OHdG was used as a competitive factor to block mitochondrial 8-OHdG to suppress the maturity and secretion of IL-1β and IL-18 (13). These findings provide strong evidence that oxidative mtDNA may be a direct signal to initiate the NLR-mediated innate immune response in dry eye (13).

### Extracellular DNA and Neutrophil Extracellular Traps May Be Involved in Dry Eye

In addition to nuclear and mitochondrial DNA, extracellular DNA fragments (eDNA) and neutrophil extracellular traps (NETs) can be involved in the pathogenesis of dry eye as autologous DNA. eDNA is any DNA that an organism releases into the environment. NETs are special structures that form after neutrophil necrosis or apoptosis and consist of nucleic acid materials without any other cytoskeletal proteins. Both eDNA and NETs are types of autologous DNA. eDNA is released from the intracellular to extracellular space during apoptosis or necrosis (73, 86). Large amounts of eDNA have been found on the ocular surface of patients with severe dry eye, and it is mainly derived from shed corneal and conjunctival epithelial cells. Lack of DNase in the tears of dry eye patients leads to the accumulation of eDNA (14). Furthermore, hyperosmotic stress can induce neutrophils to release NETs (87). NETs are also detected in patients with dry eye with increased tear osmotic pressure (14). In addition, corneal fluorescein sodium staining and increased inflammatory cytokines were observed in mice treated with media containing NETs (88). According to DEWS II, more studies are needed to prove the role of eDNA and NETs in dry eye, which could be the focus of future studies (7). The involvement of eDNA and NETs in dry eye provides strong evidence for our hypothesis that DNA could contribute to the occurrence of dry eye.

### Toll-Like Receptor 9 Recognized DNA in Dry Eye

As a danger-related molecular pattern (DAMP), DNA can be recognized by pattern recognition receptors (PRRs) and activate the innate immune system (89). Toll-like receptor 9 (TLR9) is a PRR and has been demonstrated to participate in dry eye by recognizing dsDNA (90). In addition, TLR9 is a sensor that recognizes CpG fragments in bacterial or viral DNA (91). In the quiescent state, TLR9 is located in the endoplasmic reticulum. When infection occurs, the CpG DNA of bacteria or viruses can enter cells through endocytosis to bind to TLR9 (92). Studies have shown that eDNA can enter cells and bind to TLR9 (73), which can activate inflammatory pathways through MyD88 (93, 94) to initiate the type I interferon response and enhance the adaptive immune response mediated by dendritic cells (95). In the ocular surface of patients with dry eye, increased expression levels of TLR9, MyD88, INF-α, and IFN-β have been detected (14).

The role of TLR9 in the inflammatory response of dry eye indicates that DNA may initiate immune-inflammatory responses in dry eye. The possible mechanism is that eDNA is released after cell death and enters epithelial cells of the ocular surface to be recognized by TLR9 to initiate the innate immune response (7).

## The Hypothesis That cGAS-STING Pathway Can Be Involved in the Pathogenesis of Dry Eye

**Figure 2:**
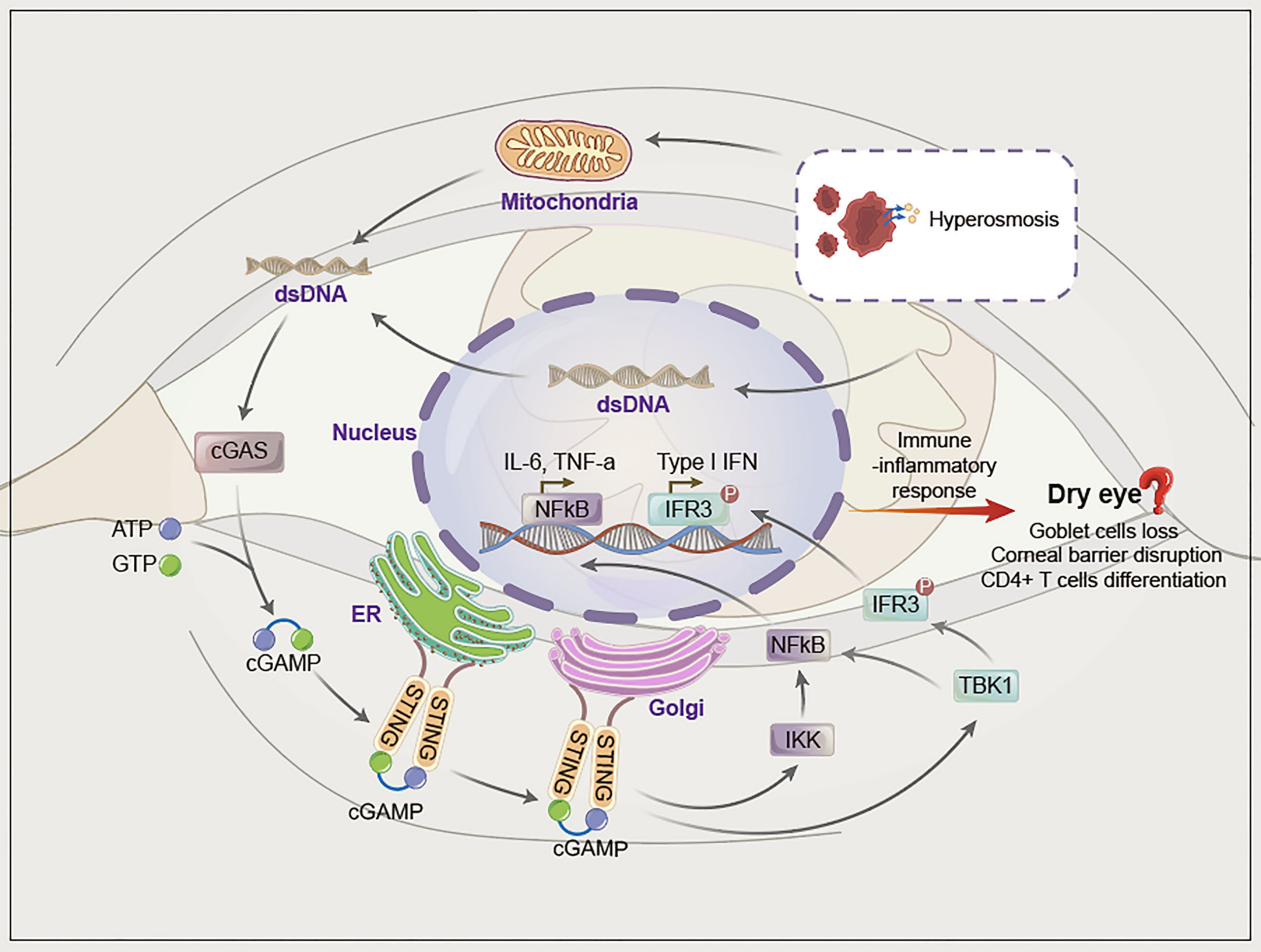
The hypothesis of cGAS-STING pathway activation in dry eye. Hyperosmotic stress induces mitochondrial and nucleafr damage, after which dsDNA (mainly mitochondrial dsDNA) is damaged and released into the cytoplasm. Cytoplasmic dsDNA is recognized by cGAS, which converts ATP and GTP into cyclic GMP-AMP (cGAMP) and activates STING. STING migrates from the endoplasmic reticulum to the Golgi apparatus and initiates inflammation *via* phosphorylation downstream of TBK1/IRF3 and NF-κB.

In summary, although there is no direct evidence that the pathogenesis of dry eye is related to the cGAS-STING pathway, when combined with the current research evidence, we propose that the cGAS-STING pathway may be a new mechanism involved in dry eye (**Figure 2**). The reasons are as follows.

First, cGAS can be activated by various endogenous/exogenous dsDNA released into the cytoplasm, whereas factors such as tear hyperosmotic stress can mediate the release of nuclear or mitochondrial DNA into the cytoplasm and activate the cGAS-STING pathway. In addition, eDNA and NETs have been reported to participate in dry eye, although the specific mechanism has not yet been elucidated. eDNA and NETs contain dsDNA, thus indicating the possibility of involvement of the cGAS-STING pathway in dry eye. Second, the cGAS-STING pathway eventually activates NF-κB and IFN-α/β, which leads to a series of immune-inflammatory responses. NF-κB and IFN-α/β are involved in dry eye. Therefore, we propose that various pathogenic factors of dry eye may activate the cGAS-STING pathway through cytoplasmic dsDNA, which further upregulates NF-κB and IFN-α/β to mediate inflammation in dry eye. Third, accumulating data suggest that autoimmune diseases are closely related to the activation of the cGAS-STING pathway, and dry eye is a common complication of autoimmune diseases such as Sjogren’s syndrome. Hence, the cGAS-STING pathway may also be involved in dry eye (**Figure 2**).

To verify our hypothesis, we will first detect the association between the cGAS-STING pathway and dry eye in mice. The mRNA and protein levels of cGAS, STING, TBK1, IRF3, NF-κB and inflammatory cytokines (INF-α/β, IL-6 and CXCL10) in the cornea of normal and dry eye mouse models will be detected by PCR and Western blot. And then, STING^-/-^ mice or topical application of C-176 (96), a STING inhibitor, will be used to assess the corneal fluorescein staining and number of goblet cells, which will provide evidence for the therapeutic potential by inhibiting cGAS-STING pathway in dry eye.

**Figure 3:**
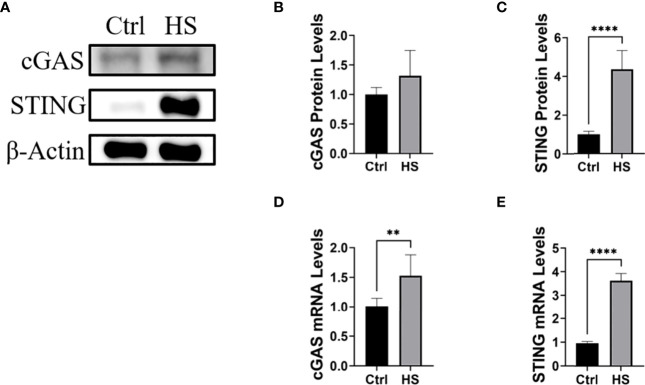
Hyperosmotic stress induced the activation of the cGAS-STING pathway in HCE. **(A-C)** The protein levels of cGAS and STING in HCE treated with hyperosmotic stress for 24h. **(D, E)** The RT-PCR results of cGAS and STING in HCE treated with hyperosmotic stress for 24h. **P < 0.01, ****P < 0.0001.

Next, HCE will be treated with hyperosmotic stress to construct an *in vitro* dry eye model. In such a model, the cGAS-STING pathway and inflammatory cytokines will be detected, which will be further verified by knock-downing cGAS or STING using siRNA. In our preliminary study, we found that hyperosmotic stress could induce the activation of cGAS-STING pathway (**Figure 3**, unpublished data).

Finally, we will detect cytoplasmic dsDNA in hyperosmotic stress-treated HCE by dsDNA staining and mitochondrial DNA PCR, which will be further verified by using ethidium bromide (EtBr), a reagent to block the replication of mtDNA.

## Conclusion and Outlook

Dry eye is one of the most common ocular surface diseases. To explore the pathogenesis of dry eye can help us better understanding and treating dry eye. Immune-inflammatory responses are the core pathologies of dry eye, and increasing evidence supports a central role for the cGAS-STING pathway in autoimmune and inflammatory diseases. Although there is no clear evidence to show whether the cGAS-STING pathway is involved in dry eye, based on the current research, we speculate that the cGAS-STING pathway may be a new mechanism that causes dry eye, and targeting the cGAS-STING pathway might be potential therapeutic strategy, which could pave the way for precision treatments and drug development of dry eye and is worthy of further study.

## Data Availability Statement

The original contributions presented in the study are included in the article/supplementary material. Further inquiries can be directed to the corresponding authors.

## Author Contributions

WO and SW wrote the article. All authors contributed to the article and approved the submitted version.

## Funding

This work was supported by The National Key R&D Program of China (2018YFA0107304).

## Conflict of Interest

The authors declare that the research was conducted in the absence of any commercial or financial relationships that could be construed as a potential conflict of interest.

## Publisher’s Note

All claims expressed in this article are solely those of the authors and do not necessarily represent those of their affiliated organizations, or those of the publisher, the editors and the reviewers. Any product that may be evaluated in this article, or claim that may be made by its manufacturer, is not guaranteed or endorsed by the publisher.
